# Supplementary figures and images for: Coordinated regulation of gene expression in Plasmodium female gametocytes by two transcription factors
Source: eLife. 2024 Jan 22;12:RP88317. doi: 10.7554/eLife.88317 (PMC10945693; doi:10.7554/eLife.88317)

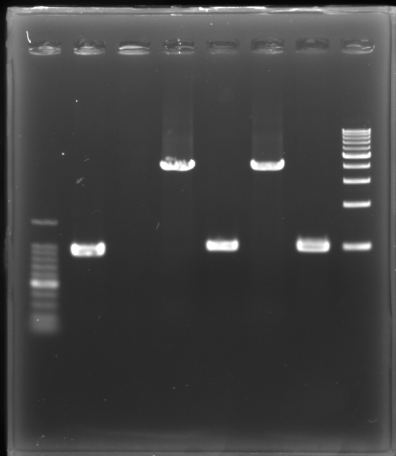

Supplement: Figure 1—figure supplement 1—source data 1. [file elife-88317-fig1-figsupp1-data1.pdf]

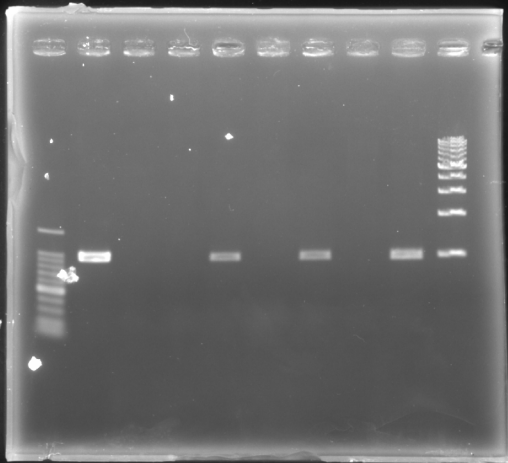

Supplement: Figure 2—figure supplement 1—source data 1. [file elife-88317-fig2-figsupp1-data1.pdf]

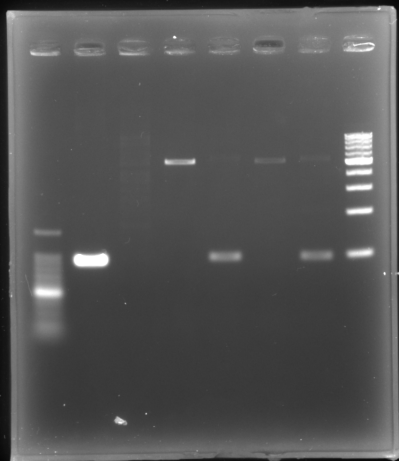

Supplement: Figure 4—figure supplement 1—source data 1. [file elife-88317-fig4-figsupp1-data1.pdf]

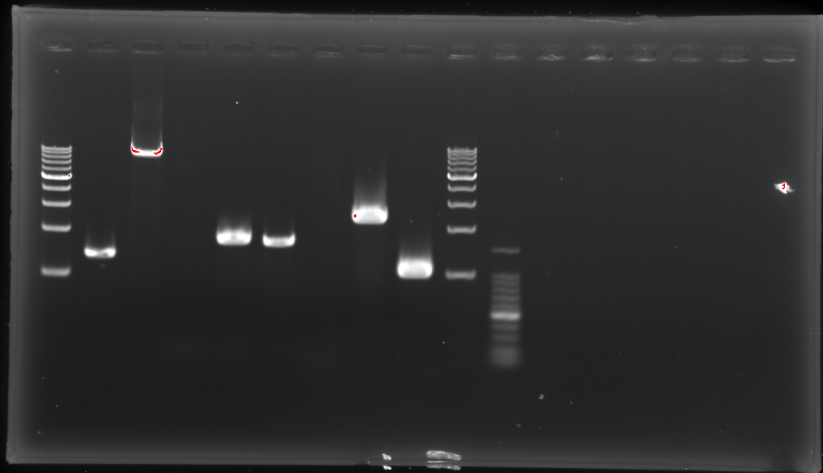

Supplement: Figure 4—figure supplement 2—source data 1. [file elife-88317-fig4-figsupp2-data1.pdf]
